# Mucosal Vaccine Development Based on Liposome Technology

**DOI:** 10.1155/2016/5482087

**Published:** 2016-12-29

**Authors:** Valentina Bernasconi, Karin Norling, Marta Bally, Fredrik Höök, Nils Y. Lycke

**Affiliations:** ^1^Mucosal Immunobiology and Vaccine Center (MIVAC), Department of Microbiology and Immunology, Institute of Biomedicine, University of Gothenburg, 40530 Gothenburg, Sweden; ^2^Department of Applied Physics, Chalmers University of Technology, 41296 Gothenburg, Sweden

## Abstract

Immune protection against infectious diseases is most effective if located at the portal of entry of the pathogen. Hence, there is an increasing demand for vaccine formulations that can induce strong protective immunity following oral, respiratory, or genital tract administration. At present, only few mucosal vaccines are found on the market, but recent technological advancements and a better understanding of the principles that govern priming of mucosal immune responses have contributed to a more optimistic view on the future of mucosal vaccines. Compared to live attenuated vaccines, subcomponent vaccines, most often protein-based, are considered safer, more stable, and less complicated to manufacture, but they require the addition of nontoxic and clinically safe adjuvants to be effective. In addition, another limiting factor is the large antigen dose that usually is required for mucosal vaccines. Therefore, the combination of mucosal adjuvants with the recent progress in nanoparticle technology provides an attractive solution to these problems. In particular, the liposome technology is ideal for combining protein antigen and adjuvant into an effective mucosal vaccine. Here, we describe and discuss recent progress in nanoparticle formulations using various types of liposomes that convey strong promise for the successful development of the next generation of mucosal vaccines.

## 1. Introduction

**Figure 1:**
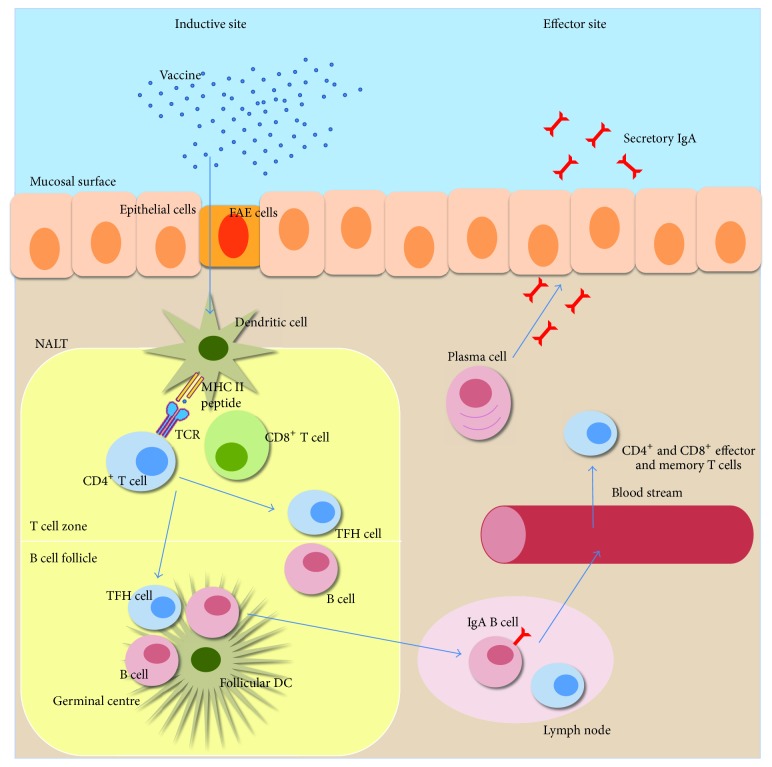
Principles for induction of mucosal immune responses after intranasal vaccination. The respiratory mucosal immune system consists of clusters of lymphoid cells beneath the mucosal epithelium, hosting both innate and adaptive immune cells [29]. There is a clear distinction between inductive and effector sites and these are also physically separated. Inductive sites are organized lymphoid tissues where antigen is taken up by DCs and other APCs. The effector sites, on the other hand, are tissues that provide protection against infection where specific antibodies and CD4^+^ and CD8^+^ effector and memory T cells reside [30]. The main inductive sites for mucosal immune responses after intranasal vaccination are known as nasopharynx-associated lymphoid tissue (NALT), which harbors B cell follicles and T cell zones in well demarked microanatomical areas [31]. Antigens are taken up by DCs that get access to the luminal content either through direct uptake through the epithelium or via the follicle associated epithelium (FAE) that overlay the NALT. After antigen uptake, the immature DCs undergo maturation and subsequently leave the mucosal tissue for the draining lymph nodes, alternatively, if already in the NALT, the DCs will directly prime naive CD4^+^ or CD8^+^ T cells. Activated CD4^+^ T cells differentiate into various subsets: T helper 1 (Th1), Th2, or Th17 cells, regulatory T cells (Tregs), or follicular helper T cells (T_FH_). The latter are critically needed for the expansion and differentiation of the activated B cells in the germinal center (GC), which is formed in the B cell follicle in the lymph node after vaccination. T_FH_ cells are involved in the development of long-lived plasma cells and memory B cells in the GC.

Most pathogens enter the body through mucosal surfaces and, therefore, vaccines that target the respiratory, gastrointestinal, or urogenital tracts are attractive as they stimulate local protection against infections. However, because of the requirements for strong mucosal adjuvants and usually relatively large amounts of antigen, only few such vaccines have been developed and most of these are live attenuated vaccines. Whereas live attenuated vaccines can be effective, subcomponent vaccines are usually safer and with less manufacturing and regulatory complications. Therefore, efforts are focused on developing mucosal vaccines based on subcomponents, but this also requires identifying appropriate and effective mucosal adjuvants to enhance the immune response. Subcomponent vaccines can consist of bacterial whole cell components, virus-like particles or other particles, polysaccharides, complete protein structures, or peptides that delivered at mucosal membranes together with an adjuvant can stimulate strong immune responses and protection against infection. Such mucosal vaccines are much warranted, as they carry several advantages over injectable vaccines. In particular, mucosal vaccines can elicit both local and systemic immune responses and they are safer as they do not require needles and may allow for mass vaccination, when pandemic spread of infection is a threat [1, 2]. Mucosal vaccination could also lead to increased compliance and reduce the risk of spreading transmissible diseases, as has been experienced with spread of hepatitis C and HIV infections following the use of injectable vaccines [3]. Most importantly, mucosal immunization elicits antigen-specific local IgA and systemic IgG antibodies, as well as strong systemic and tissue resident CD4^+^ and CD8^+^ T cell immunity (Figure 1). Despite these advantages, only few mucosal vaccines are commercially available. The reason for this is the need for safe and effective mucosal adjuvants and the fact that many vaccine formulations require protection from degradation of the antigens as seen, for example, after oral administration [4]. Consequently, the development of novel combinations of antigen and adjuvant into nanoparticles for the next generation of effective mucosal vaccines is much needed.

Liposomes have been extensively used as delivery vehicles for vaccine antigens; some of the advantages of these formulations are (a) protection against antigen degradation, (b) tissue depot effects or slow release of antigen, and (c) facilitated uptake of antigen by antigen presenting cells (APC) [5, 6]. Phosphatidylcholines are the most common lipids employed for liposome manufacturing. However, nanoparticles can be developed from a wide range of lipids and proteins, which have been found to also alter their physicochemical and biological properties. Classical liposomes are now also gradually being replaced by more advanced technologies with the new generation of lipid-based nanovesicles (L-NVs), which have more elaborate functions and less weaknesses. Niosomes, transfersomes, sphingosomes, and other nonliposomal lipid-based nanoparticles are excellently reviewed by Grimaldi et al. [7]. For example, the virus-like particle (VLP) or virosome L-NV incorporates virus-derived or recombinant proteins that in this way are effectively delivered to the immune system [8]. This technology is used in two commercial vaccines, Inflexal (against influenza) and Epaxal (against hepatitis A) [9, 10]. Currently, several liposome-based vaccine delivery systems against infectious diseases are undergoing clinical testing (Table 1).

Mucosal vaccines were initially designed to be administered orally. Later, also intranasal vaccines were developed and today several different routes of administration are being explored for mucosal vaccination, including pulmonary, genital tract, rectal, and sublingual routes. Whereas preclinical examples in animal models have shown that in principle all of these routes work well, only the oral and intranasal routes have been used for licensed human vaccines [11]. The reason for this may simply be attributed to that a majority of these vaccines are against gastroenteric infections (requiring oral vaccines). Importantly, the adjuvant choice is critical because it enhances and modulates the immune response to the vaccine. For example, the breadth, the quality, and the long-term protective effect of the vaccine may be directly dependent on the adjuvant [12]. Liposomes can also function as adjuvants in their own right, as they have been shown to enhance immune responses even after oral administration [13]. A particular type of liposomes, that is, layersomes, which are liposomes coated with single or multiple layers of biocompatible polyelectrolytes, has been found to stimulate significant serum IgG and mucosal IgA antibodies and T cell responses producing IL-2 and IFN-*γ* [14]. Noteworthy, though, the oral route most often requires high amounts of antigen and protection against enzymatic degradation. Moreover, an effective oral liposome vaccine should be effective at breaching the mucus barrier to facilitate uptake of antigen by gut mucosal antigen presenting cells (APCs).

Whereas oral vaccination provides a real challenge to vaccine developers, the intranasal (i.n.) route is more permissive. In fact, i.n. vaccination has several advantages compared to oral vaccination. These include the need for less antigen and a substantially reduced risk of antigen degradation [15]. Interestingly, IgG-coupled liposomes with an enhanced transmucosal transport were more immunogenic than plain liposomes given i.n. [16]. Because of the compartmentalization of the mucosal immune response, due to the acquisition of tissue-specific homing receptors on activated lymphocytes, nasal immunization also promotes a much stronger specific immune response in the respiratory tract compared to oral immunization. This also results in that excellent genital tract immunity can be achieved after i.n, immunizations, while this is not the case after oral vaccination. Thus, local secretory IgA (sIgA) antibodies and genital tract cytotoxic T cells were more effectively stimulated after i.n. immunization than through oral immunizations [17–20].

In this review we will describe and discuss liposomes as vaccine delivery vehicles for efficient mucosal immunization. In the first section, the impact of liposome composition and structure for vaccine efficacy will be discussed and in the second section the nature and qualities of the resulting immune responses following liposome vaccination will be described. Finally, in the last section, we have attempted to summarize the current standing of the field of liposome-based vaccines and future perspectives towards the development of the next generation of effective mucosal vaccines.

## 2. Liposomes as Vaccine Delivery Vehicles

**Figure 2:**
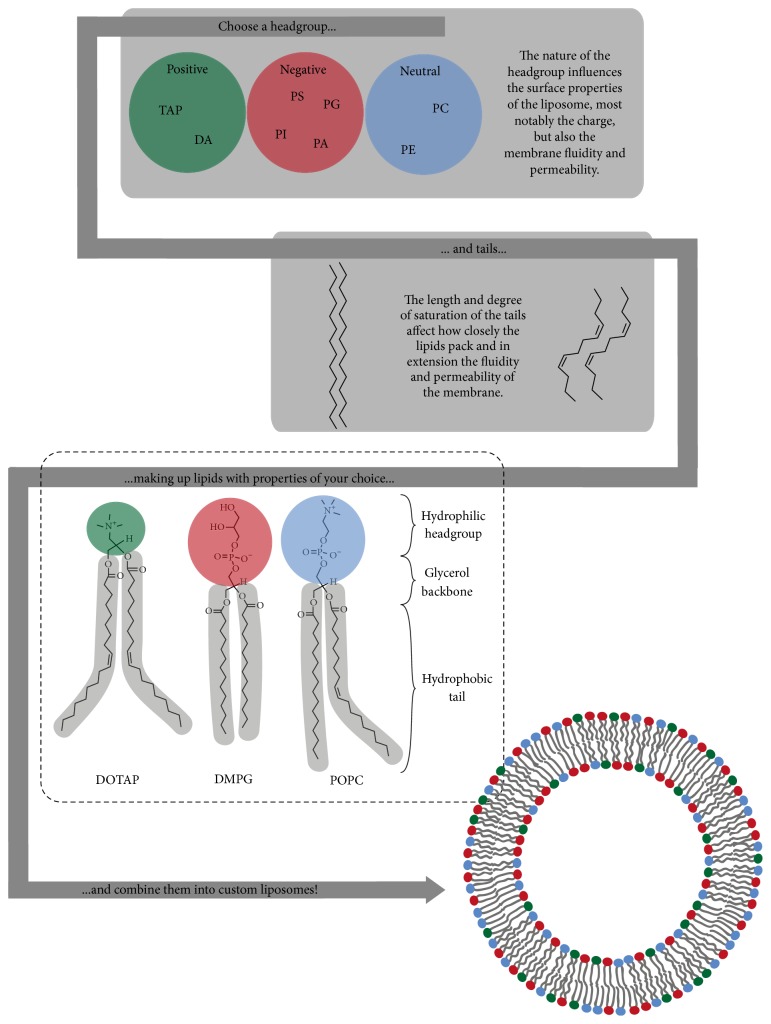
Generation of customized liposomes. Lipids are polar molecules consisting of a hydrophilic headgroup and hydrophobic fatty acid tails. Examples of positively charged headgroups are trimethylammonium propane (TAP) and dioctadecyl ammonium bromide (DA), while negatively charged headgroups are phosphatidylserine (PS), phosphatidylinositol (PI), phosphatidylglycerol (PG), or phosphatidic acid (PA), and finally neutral headgroups are phosphatidylcholine (PC) or phosphatidylethanolamine (PE). A headgroup can be combined with tails of different nature to create lipids with the desired properties; the examples shown are the lipid 1,2-dioleoyl-3-trimethylammonium propane (DOTAP) and the phospholipids dimyristoylphosphatidylglycerol (DMPG) and 1-palmitoyl-2-oleoyl-sn-glycero-3-phosphocholine (POPC). Different lipids can then be combined into liposomes with different functional features, which provide the basis for this highly diverse and versatile technology that is so excellently suited for vaccine development.

Liposomes are spherical lipid bilayer structures with an aqueous core ranging in size from tens of nanometers to several micrometers in diameter. Liposome technology was first explored in the 1960s by Bangham et al. as a model system for diffusion of ions across biological membranes, and already in the 1970s there was an interest in using them for drug delivery [39, 40]. At that time, some researchers also tested them for adjuvant functions and ever since they have been used in various vaccine formulations owing to their inherent structural and chemical properties [41]. Phospholipids are most commonly the main constituents of the shell that delimit the aqueous core of the liposome. These molecules are amphiphilic and contain a hydrophobic tail consisting of two fatty acids linked by a glycerol backbone to a hydrophilic headgroup made up of phosphate and potentially another organic molecule that together determine their chemical properties, as shown in Figure 2. In an aqueous environment, this polarized structure facilitates self-assembly into arrangements with the fatty acids facing each other and forming an oil-like compartment between the outwards-facing phosphate groups. In liposomes, the arrangement is a hollow sphere, a vesicle consisting of either a single or multiple phospholipid bilayers forming unilamellar or multilamellar particles. Other types of lipids, all with the amphiphilic structure in common, may be incorporated in liposomes. Examples of other interesting lipid categories in the context of vaccine carrier formulations are sterols, most notably cholesterol (Chol); sphingolipids, with a sphingosine backbone linked to a headgroup and a single acyl chain tail; and fat-soluble vitamins such as tocopherols (vitamin E).

**Figure 3:**
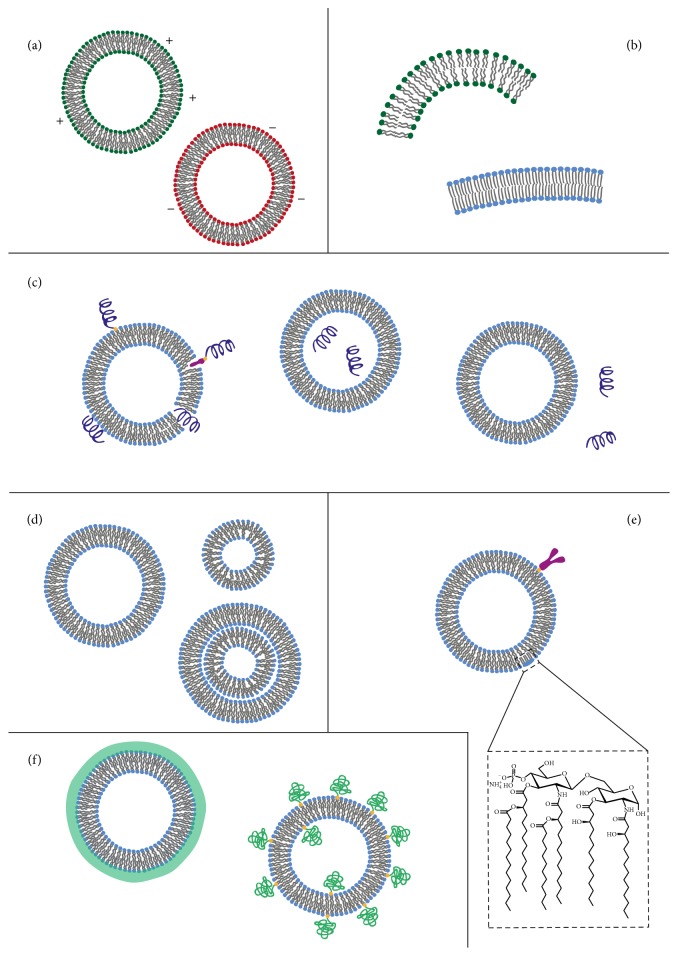
Various properties of liposome-based vaccines. (a) The effect of altered surface charge on liposome function has been extensively examined [32, 33]. (b) The lipid composition is critically influencing the immune response [34]. (c) Also the localization of the antigen on or inside the liposome plays an important role in shaping the immune response to the vaccine. There are several modes of antigen association to liposomes. Firstly, antigens may be encapsulated in the aqueous core or they could be linked to the surface via covalent attachment. Alternatively, a hydrophobic anchor can be used to attach the antigen to the surface via adsorption or through electrostatic interactions with lipids of opposite charge. For proteins with a hydrophobic region one may even successfully insert these in the liposome membrane. The liposome may also be used as an immunoenhancer simply by admixing the antigen and the liposomes. (d) Only few studies have addressed the impact of size or lamellarity [35, 36]. (e) Modifications of liposomes to increase their immunoenhancing effect can be done through attaching PAMPs, such as lipid A (LPS), or through specific targeting strategies using cell-specific antibodies (anti-CD103 or -DEC205) [16, 37]. (f) Other modifications, including addition of poly(ethylene glycol) (PEG) or different polymer coatings that increase the liposome penetration of the mucosal barrier or to increase liposome resistance in biological fluids, have also been developed [38].

The fact that liposomes have both a hydrophobic and a hydrophilic region makes them versatile and useful as carriers for antigens: hydrophobic peptides or proteins can be inserted into the inner hydrophobic center of the bilayer while hydrophilic molecules can be either encapsulated in the core of vesicles or bound to their surface (Figure 3(c)). Surface binding of the antigen can be achieved by covalent attachment or it can occur through adsorption or electrostatic interactions. Alternatively, an antigen-bound hydrophobic anchor can be inserted into the bilayer. Another advantage of liposomes in vaccine formulations is that their physicochemical properties are highly adaptable and their size, charge, and lamellarity can be tailored to meet the requirements of the vaccine. In particular, the charge and membrane fluidity of the liposome can be fine-tuned by altering the lipid composition [42].

The properties of different phospholipids depend on both their polar headgroup and the nature of their fatty acid tails. The characteristics of the lipid headgroup play a key role in determining the surface properties and in particular the surface charge of the vesicles. Naturally occurring phospholipids can be categorized into 6 types according to their headgroup: phosphatidylcholine (PC), phosphatidylethanolamine (PE), phosphatidylserine (PS), phosphatidylinositol (PI), phosphatidylglycerol (PG), or phosphatidic acid (PA). Whereas PS, PI, PG, and PA are negatively charged, PC and PE are neutral but zwitterionic. Further, both the fluidity and permeability of the membrane and, in extension, its resistance to degradation, here referred to as stability, depend both on the length and degree of saturation of the acyl chains of the tail as well as the charge of the headgroup. All of these factors influence the transition temperature of the lipid, which is in turn decisive for whether the lipid membrane exists in gel- or fluid-phase at a certain temperature. These factors also determine the tendency of multicomponent membranes to undergo small-scale phase separations resulting in heterogeneous distribution of different lipids. A common modulator of the membrane permeability and fluidity is cholesterol, which also influences the liquid-to-gel phase temperature [43].

The possibility to chemically modify both the headgroup and the tail region gives rise to the option of producing synthetic phospholipids tailored to specific requirements. For example, positively charged liposomes have been made using synthetic cationic lipids, such as 1,2-dioleoyl-3-trimethylammonium propane (DOTAP), 1,2-dimyristoyl-trimethylammonium propane (DMTAP), and dimethyldioctadecylammonium bromide (DDA). From this, it follows that the choice of lipid composition will greatly impact the biophysical and chemical properties of the liposome. Also, by choosing lipids that naturally exist in cell membranes, liposomes can be completely biodegradable, nontoxic, and nonimmunogenic in themselves [44, 45]. On the other hand, if components with an origin in archaeal, bacterial, or viral membranes are chosen, they could enhance the immunogenicity of the formulation [35, 37, 46–50]. Accordingly, PS is naturally exposed on the surface of cells undergoing apoptosis, and in this way liposomes containing PS may effectively trigger phagocytosis by macrophages [51–53]. Finally, many other modifications can be made to liposomes that further extend the high versatility of these nanoparticles. Such modification includes the attachment of targeting agents such as galactose or APC-specific antibodies, of polymers, for example, poly(ethyleneglycol), or the addition of different kinds of coatings such as chitosan [38, 54–58]. Furthermore, when introduced into a physiological fluid, nanoparticles such as liposomes acquire a dynamic layer of adsorbed proteins in a corona, the nature of which is determined by both the particle's size and surface properties as well as the shear stress the particle is exposed to [59–62]. In practice this means that carefully engineered surface properties may be altered by the adsorption of a complex protein mixture. This can have many diverse consequences; for example, the protein corona may mask surface bound ligands and in this way prevent receptor binding, or it can cause complement activation [63–65].

## 3. Physiochemical Properties of Liposomal Vaccines

### 3.1. Surface Charge

Although liposomes with different characteristics have been extensively used for vaccine delivery, it still remains to explain why the immune response is modulated differently by different liposomal formulations. It is inherently difficult to dissect the contribution of different properties, as changing one property usually influences one or several others. Surface charge can, for instance, be changed by altering the lipid composition; however, by changing the lipid composition also other properties might change, such as membrane fluidity, rigidity, and stability. Hence, it may be difficult to directly assess the influence of changing different physicochemical properties of liposomes on the immune response. Nevertheless, many attempts have been made to describe the effects on immune responses after altering liposomal characteristics. One of these parameters is the charge of the liposome, which is assessed by the zeta potential, a measure of the electrostatic potential at the limit of what is called the diffuse electric double layer that surrounds the particle (Figure 3(a)). The double layer is a diffuse layer of differently charged ions spatially distributed at the surface of the particle, which in this way becomes shielded. The magnitude of the zeta potential, thus, depends on the concentration of ions within the double layer, but also other factors, such as the ionic strength and pH of the dispersion medium. This must be kept in mind when comparing zeta potential values reported in different studies and under different conditions.

Because the cell surface as well as the mucus coating of the mucosal membrane is negatively charged, it is frequently hypothesized that positively charged liposomes will exhibit stronger interactions with the cell membrane as well as an increased mucoadhesion. The latter leads to reduced clearance rate, that is, slower removal from the mucosal membranes. Noteworthy, both increased interactions with the cell membrane and prolonged exposure time of the antigen at the mucosal surface are thought to lead to increased cellular uptake of antigen and stronger immune responses. However, this may not always be the case. In general, positively cationic charged liposomes have been shown to be better retained and more immunogenic at mucosal membranes than negatively charged or neutral liposomes [66, 67]. Furthermore, cationic liposomes were found to effectively deliver antigen to both mucus and antigen presenting cells (APCs) as shown in an in vitro model of the airway epithelium with liposomes made with distearoylphosphatidylcholine (DSPC)/trehalose 6,6-dibehenate (TDB) (neutral) and DSPC/TDB/DDA (positive) with varying amounts of DDA [68]. Moreover, cationic liposomes consisting of DOTAP/Chol, DMTAP/Chol, or, most prominently, the polycationic sphingolipid ceramide carbamoyl-spermine (CCS) and cholesterol were shown to effectively stimulate systemic and mucosal humoral and cellular immune responses after i.n. immunizations in mice [32]. By contrast, neutral liposomes with dimyristoylphosphatidylcholine (DMPC) or anionic liposomes with DMPC/dimyristoylphosphatidylglycerol (DMPG) were comparably ineffective as immunogens [32]. While a positive charge appears to increase the immunogenicity of liposomes, it still remains to be investigated in greater detail. In fact, negatively charged liposomes have been shown to be more immunogenic than both zwitterionic and positively charged liposomes and it has even been postulated that anionic liposomes could exert an immunosuppressive effect on alveolar macrophages and in this way promote an enhanced humoral immune response [33, 69–72]. Hence, it can be hypothesized that several mechanisms are modulated by the charge of the liposome. It is also important to point out that altered charge of the liposomes by necessity involves modifying the lipid composition, which most likely will change also other properties, such as membrane heterogeneity, fluidity, and stability [73]. Naturally, also the charge of the liposome may dramatically influence the immunogenicity when given by different routes, as this may provide differentially charged microenvironments.

### 3.2. Lipid Composition

The lipid composition (Figure 3(b)) is known to influence the stability of the liposome; a more stable formulation might lead to a larger amount of bioavailable antigen and potentially also a depot effect. Han et al. made liposomes from various combinations of Chol, dipalmitoylphosphatidylcholine (DPPC), dipalmitoylphosphatidylserine (DPPS), and distearoylphosphatidylcholine (DSPC) and found that certain combinations impacted on their stability. Liposomes with DSPC, having a higher transition temperature, were more stable in vitro and likely protected antigen better from degradation in the gastrointestinal tract [49]. It was also found that stable liposomes containing DPPS induced stronger IgA responses compared to formulations without DPPS [34]. Combinations of both DPPC/DMPG and DPPC/PS have been found effective at targeting liposomes to macrophages, though DPPC/DMPG were found more immunogenic than liposomes with DPPC/palmitoyl phosphatidylethanolamine (DPPE) or DPPC/PS. Noteworthy, changing the lipid composition also resulted in an altered charge, with the DMPG and PS types being more negatively charged [73]. Furthermore, cationic liposome-hyaluronic acid (HA) hybrid nanoparticle systems have recently been developed and tested for DC maturation. It was found that primarily an upregulation of costimulatory molecules, including CD40, CD86, and MHC class II, were responsible for an enhanced effect, which greatly contributed to an enhanced specific T cell and antibody response following i.n. vaccination [74].

As previously mentioned, using archaeal lipids, liposomes can be made more immunogenic and liposomes comprising archaeal membrane lipids (archaeosomes) were found considerably more potent than liposomes made with Egg phosphatidylcholine (EPC)/Chol at inducing ovalbumin- (OVA-) specific IgG and IgA antibodies following oral administration in a mouse model [46]. This was likely due to increased stability in the gastrointestinal tract and to the fact that the archaeosomes were better retained in the intestine [46]. However, the difference may also partly reflect the fact that the archaeosomes are negatively charged while the EPC/Chol-liposomes are neutral.

### 3.3. Antigen Localization in Liposomal Formulations

There are many ways of incorporating antigens into liposomes. This raises the question of whether some strategies are more effective than others in the context of optimizing the immunogenicity of the liposome. Antigens can be hosted in the aqueous core of the liposome, inserted into the membrane leaflet or bound to the surface by covalent bonds or intermolecular forces (Figure 3(c)). Hence, a plethora of combinations exist and those could be used to enhance resistance against antigen degradation or facilitate antigen uptake. Thus, the liposome formulation may be tailored for specific needs and purposes. If an oral vaccine is to be designed, one may hypothesize that encapsulating the antigen inside the liposomes should be an effective strategy to prevent enzymatic degradation. However, by hiding the antigen in the liposome, the immunogenicity may be compromised because the antigen will not be immediately accessible for APCs. Therefore, choosing how to physically incorporate the antigen in the liposome may have critical consequences and could dramatically change the immune response. Unfortunately, until now such aspects have not been addressed in a systematic manner. When administered orally, encapsulated antigen may more effectively stimulate local IgA and serum IgG antibody responses compared to when soluble antigen is admixed with the liposomes [73, 75]. On the other hand, following i.n. administration, admixed antigen and liposome have been quite effective even compared to liposome-encapsulated antigen [32, 70]. Interestingly, liposomes have been found to exert an immunoenhancing effect even when administered 48 hours prior to the antigen [70]. Furthermore, even liposome surface bound antigens, rather than fully encapsulated antigens, have been found to be more immunogenic following i.n. immunization [71]. Probably, these observations underscore that the i.n route is less sensitive to antigen degradation compared to the oral route. Thus, depending on the route of administration, it seems clear that antigens may or may not be immunogenic when exposed on the surface of the liposome and for many formulations it may, in fact, be advantageous to have surface bound as well as encapsulated antigens. Indeed, this may also apply to the adjuvant. It was found that cholera toxin B-subunit (CTB) adjuvant bound to the surface of the liposome was more effective compared to when encapsulated in the liposome [76]. In fact, a challenging question is what the relationship and localization should be between the antigen and the adjuvant in the liposome. Theoretically, it can be argued that since the adjuvant is included primarily to promote dendritic cells- (DC-) priming of the T cells it should be encapsulated, while the antigen should be both encapsulated and surface bound to secure sufficient stimulation also of naïve B cells. Of note, B cells normally recognize 3D structures with their receptors, while T cell receptors react to degraded linear peptides. However, this interesting question has been poorly investigated and only few studies have been published on this topic. For example, it has been observed that by altering the lipid-to-antigen ratio, the humoral and cellular immune responses can be differentially induced [32, 77]. Thus, it is likely that the immune response following liposome administration is susceptible not only to the choice of antigen and lipid components but also to their relative proportions and localization in the liposome.

### 3.4. Size and Lamellarity

A broad range of unilamellar and multilamellar liposomes with varying sizes have been found to have variable effects following mucosal immunization (Figure 3(d)). While, unfortunately, the degree of multilamellarity is not routinely reported, the influence of size and/or lamellarity on the immunogenicity of the liposome is yet to be determined. For example, a comparative study between unilamellar archaeosomes, 100 nm in diameter, or large multilamellar aggregates of these clearly identified better immunogenicity of the multilamellar aggregates [35]. Noteworthy, not only the size but also the lipid assembly was different between the unilamellar and multilamellar constructs, in this example. On the other hand, another study reported that a “double liposome,” consisting of small (~250 nm) liposomes made from SoyPC, DPPC, Chol, and SA encapsulated into a bigger (1 to 10 *μ*m) outer liposome made from DMPC and DMPG, was found only marginally more immunogenic than the small liposomes given alone by oral administration [36]. Taken together, constructing homogeneous monodisperse and unilamellar liposomes is highly challenging and various degrees of multilamellar constructs may coexist, making interpretations of experimental results difficult, but recent advancements in this technology may allow for more accurate comparisons of the influence of size, lamellarity, and overall structure in the future [78].

### 3.5. Modifications Increasing the Bioavailability of Liposomal Antigens

The microenvironment at mucosal surfaces often promotes a high clearance rate of liposomes. Therefore, various strategies have been tested to enhance mucus penetration or to increase membrane adhesion to facilitate bioavailability of the vaccine antigens (Figure 3(f)). Layer-by-layer deposition of polyelectrolytes onto the liposome, for example, has been used as a liposome-stabilizing approach which resulted in higher specific IgA and IgG antibody levels as well as an increased T cell response [79]. Polyvinyl alcohol or chitosan has been tested to enhance bioadhesive properties of the liposome and it has been observed that chitosan-loaded liposomes, indeed, stimulated enhanced IgG antibody responses [58]. Chitosan is a positively charged polysaccharide that can form strong electrostatic interactions with cell surfaces and mucus and, therefore, increase retention time and facilitate interactions between the liposome and APCs in the mucosal membrane. Alternatively, such modifications can also transiently open tight junctions between epithelial cells to allow for transmucosal transport of the liposomes [80–82]. In fact, chitosan-coated liposomes have been shown to give better serum IgG antibody levels compared to other bioadhesive polymers, such as hyaluronic acid or carbopol coated liposomes, and host much better immunogenicity than uncoated negative, neutral, or positively charged liposomes [38, 56–58].

Considerable attention has been given to studying how liposomes are retained by and/or taken up across the mucosal membranes. Liposome interactions with the intestinal mucosa have been studied in vivo and ex vivo using various in vitro models [46, 79, 83, 84]. The latter models have addressed whether passage of liposomes between the tight junctions of epithelial cells can be achieved. Indeed, tight junctions were reported to be open when using PC/Chol-liposomes or* Tremella*-coated liposomes [84]. Enhanced immune responses were also observed with mucus-penetrating liposomes made with poly(ethylene glycol) (PEG) or the PEG-copolymer pluronic [38]. Significantly higher specific IgA and IgG antibody levels were found with PEGylated than un-PEGylated liposomes. Charge-shielding modifications with PEG or Pluronic F127 also proved useful in preventing liposome aggregation to obtain small (<200 nm) chitosan-coated liposomes. In fact, these shielded chitosan-coated and PEGylated liposomes yielded the highest functional serum antibody titers of all the formulations tested and the strongest IgA responses [38].

### 3.6. Cell-Targeting Modifications of Liposomes

Modifications aimed at increasing liposome stability and/or uptake have indeed proven effective. One of the most explored modifications is aimed at targeting the delivery of liposomes to subsets of cells. Liposomes can be equipped with various targeting elements, aiming at enhancing their immunogenicity (Figure 3(e)). For example, additional targeting components may enhance the uptake by APCs or the penetration of the liposome through the mucus layer. The strongly GM1-ganglioside-binding molecule CTB has been reported to enhance liposome immunogenicity. Moreover, when monophosphoryl lipid A, acting through the TLR4 receptors, was added to liposomes their ability to stimulate the innate immune response was dramatically improved [34, 37, 49, 85, 86]. Other Toll-like receptor agonists or* Escherichia coli* heat-labile toxin (LT) have also been used in combination with liposomes as adjuvants [47, 87]. Furthermore, linking CpG, which acts through TLR9 signaling, or* Bordetella pertussis* filamentous hemagglutinin to the liposome has all been found to enhance immunogenicity [88, 89].

In fact, many different liposome cell-targeting approaches have been investigated. To this end, specific antibodies have been found to enhance binding to M cells, thereby targeting the liposome to the follicle associated epithelium (FAE). This is the thin epithelial cell layer that is responsible for antigen uptake from the luminal side, such as the epithelium that overlays Peyer's patches (PP) in the small intestine [16]. Similarly, lectin Agglutinin I from* Ulex europaeus *coated liposomes were shown to improve M cell-targeting and antigen uptake [83, 90, 91]. Also, galactosylation of liposomes resulted in higher specific IgA and IgG antibody levels compared to unmodified liposomes [54]. Moreover, liposomes coated with the influenza virus protein hemagglutinin were more immunogenic than uncoated liposomes [50]. In addition, mannosylated lipids or anti-CD40 antibody-coated liposomes were found to host an enhanced ability to target DCs and, thereby, greatly promoted a stronger immune response [55, 87]. Furthermore, the identification of Mincle, a receptor for the mycobacterial cord factor trehalose 6,6′-dimycolate (TDM), on innate immune cells, led to that TDM analogs were found to be effective stimulants of the production of G-CSF in macrophages. Indeed, immunizations in mice with cationic liposomes containing the analogues TDM demonstrated a superior adjuvant activity [92].

Many strategies have, indeed, been applied to achieve cell-targeting of liposomes, with varying degrees of improved function. Needless to say, there exist a plethora of possibilities to explore when it comes to targeting liposomes to the cells of the mucosal immune system. If analytical tools are combined with suitable in vitro and in vivo assay systems it will greatly help identifying the relative importance of liposome targeting and how composition, such as size, lamellarity, surface charge, and fluidity of the membrane, can influence the immune response.

## 4. Mucosal Immune Responses to Liposomes

Prior to an adaptive immune response, innate immune activation must have occurred, leading to the production of proinflammatory molecules and the expression by APC of costimulatory and immunomodulating molecules, that is, chemokines, cytokines, and the costimulatory molecules CD80, CD86, CD40, and others. Innate immune activators can be classified into several categories, including the dominant ones, Toll-like receptors (TLRs), C-type lectin receptors (C-LRs), and non-Toll-like receptors (NLRs) [93, 94]. These receptors recognize pathogen-associated molecular patterns (PAMPs), such as bacterial cell-wall components (e.g., peptidoglycan, lipoteichoic acid, and flagellin) and different forms of microbial nucleic acids (e.g., double-stranded RNA, high-CpG-content DNA). The role of an adjuvant in a vaccine formulation is, thus, to activate innate immunity and, therefore, most vaccine adjuvants are derived from PAMPs. Also for mucosal vaccines it is critical to induce a sufficient and appropriate innate immune response, preferentially, without causing unwanted side effects, such as tissue damage. Thus, a successful mucosal vaccine must be capable of inducing not only an adaptive immune response, but also a strong innate immune response [13]. Fortunately, liposomes can do both. They can both serve as delivery vehicles for vaccine antigens and act as immunomodulators, triggering both innate and adaptive immune responses. Indeed, many modifications of the liposome itself can dramatically influence the innate immune response and, thereby, augment or qualitatively modify also the adaptive immune response. To this end, altered lipid composition, charge, particle size, or added targeting elements can all be utilized to tailor the immune response to the liposome and stimulate the required anti-infectious immune response (Figure 3). In addition, to get an even stronger activation of innate immunity, liposomes can be equipped with specific adjuvants/PAMPs, such as flagellin or CpG, as we have already discussed.

Mucosal immunizations as opposed to systemic immunizations effectively support IgA class switch recombination (CSR) and production of sIgA at mucosal sites [95]. In the intestine, this occurs in the organized gut associated lymphoid tissues (GALT) and, in particular, in Peyer's patches (PP), which are the dominant sites for IgA CSR in the gut. In the upper respiratory tract the most active inductive site is the nasal associated lymphoid tissues (NALT), but also cervical lymph nodes and the mediastinal lymph node (mLN) are central to the mucosal immune response. Ultimately, targeting of the liposome to these sites could be a much preferred strategy in future vaccine formulations, as already discussed. Following antigen recognition and activation of specific B cells in GALT and NALT, these cells undergo strong expansion in the germinal centers (GC) in the B cell follicles. Most immune responses are T cell dependent and, thus, the expanding B cells require the participation of follicular helper T cells (T_FH_) in the GC to differentiate into long-lived plasma cells and memory B cells. These CD4+ T cells are generated through the peptide-priming event that the DCs orchestrate in the T cell zone in the lymph node. In this way, the DC is a key player in the immune response and it impacts also the ability of the activated lymphocytes to migrate to the effector tissue from where the DC originated. The plasma cells, thus, eventually migrate from the inductive site via the lymph and blood back to the lamina propria in the mucosal membrane, where they produce sIgA. Thus, there is a complex series of events that need to be completed before a productive sIgA response can be found in the lamina propria of the mucosal membrane (Figure 1). Hence, liposomes can be made to impact or modulate many different steps in this series of events. A critical question is how we can assess and compare different liposome formulations for their efficacy and characteristic impact when multiple steps are involved. We would advocate a reductionist approach where different liposomes are evaluated for their effect in different stages of the buildup of an immune response. Therefore, in the next section we focus on the interaction of liposomes with DCs and APCs in general.

### 4.1. Innate and Adaptive Immunity against Liposome Vaccination

Although significant progress has been made in understanding antigen uptake and processing of liposome delivered antigens, the fine details of these processes are still poorly known. Liposomes that enhance cell membrane fusion, that is, fusogenic liposomes, deliver their antigenic content to the cytoplasm of the APC, which enables MHC class II presentation to CD4^+^ T cells and in some DC subsets also allows for cross-presentation to MHC class I restricted CD8^+^ T cells. Of note, liposomes that are taken up via scavenger receptors (CD68, CD36, and Clec LOX-1) or other innate immune receptors are usually restricted to prime CD4^+^ T cells through MHC class II presentation. Thus, targeting of liposomes to different DC subsets or uptake mechanisms can provide a means to specifically tailor the immune response to a certain antigen or facilitate the development of a distinct type of immune response [96, 97]. For instance, whereas zwitterionic or anionic liposomes have not been reported to drive inflammation, cationic liposomes have been shown to stimulate proinflammatory responses in DCs, leading to an upregulation of costimulatory molecules, CD80 and CD86, and proinflammatory cytokines [98]. Furthermore, Yan et al. reported that DC stimulation by cationic liposomes composed of DOTAP (1,2-dioleoyl-3-trimethylammonium propane) also stimulated reactive oxygen species (ROS), which activated extracellular signal-regulated kinase (ERK) and p38, and downstream proinflammatory cytokines/chemokines, interleukin-12 (IL-12), and chemokine (C-C motif) ligand 2 (CCL2) [99]. In addition, DOTAP liposomes were shown to induce transcription of monocyte chemoattractant protein-1 (MCP-1/CCL2), macrophage inflammatory protein-1 alpha (MIP-1*α*/CCL3), and macrophage inflammatory protein-1 beta (MIP-1*β*/CCL4) [100]. Also DiC14-amidine cationic liposomes can induce the secretion of IL-1*β*, IL-6, IL-12p40, interferon-*β* (IFN-*β*), interferon-*γ*-inducible protein 10 (IP-10), and TNF-*α* by human and mouse myeloid DCs [101]. While anionic liposomes in general are poorly proinflammatory, modifications such as using mannosylated lipids could make these liposomes much more proinflammatory and effective at stimulating DCs [102, 103]. With regard to macrophages it has been reported that galactose-modified liposomes can stimulate TNF-*α* and IL-6 production, which was associated with significantly higher specific sIgA antibody levels in the nasal and lung tissues and increased serum IgG antibodies [54].

Carefully analyzing the literature, it appears unclear how different liposomes stimulate strong innate immune responses. A high density of positive charges on liposomes is considered beneficial, while negatively charged or neutral lipids are likely to lower this capacity [104, 105]. As mentioned previously, liposomes effectively stimulate both T and B cell responses, but it is their direct impact on the DCs that matters for the adaptive immune response. In fact, the uptake of liposomes by DCs has an important effect on the development of different CD4+ T cell subsets. These CD4^+^ T cell subsets have both distinct and overlapping functions, but their individual impact on the immune response is critical. For example, if protection against a pathogen requires IFN-*γ* production (Th1 cells), then the development of exclusive Th2-dominated responses can be detrimental. Therefore, in this context, monophosphoryl lipid A (MPL) (a TLR4 ligand) or monomycoloyl glycerol (MMG) combined with DDA in a liposome will consistently promote IFN-*γ* production, that is, a Th1-biased immune response [106]. Moreover, trehalose dibehenate (TDB) liposomes, together with the combined Ag85B-ESAT-6 vaccine antigen, enhanced antituberculosis specific IFN-*γ* and IL-17 production, as well as increased specific serum IgG2 antibody levels [107]. While the composition of lipids matters for the subset of CD4^+^ T cell that the DC can prime, also a larger size of the liposome may influence the generation of Th1 CD4^+^ T cells [108]. However, the mechanism for this effect is unclear but could relate to the fact that different subsets of DCs or other APCs like macrophages are involved in processing differently sized liposomes, such that small sized liposomes preferentially stimulate Th2 CD4^+^ T cell responses. On the other hand, it has been claimed by many investigators that protection is best achieved with a balanced Th1/Th2 response. Liposome-based microneedle array (LiposoMAs) and a mannose-PEG-cholesterol (MPC)/lipid A-liposome (MLLs) system are both examples of liposome formulations with a balanced Th1/Th2 response [109–111]. An even more complex vector is the combination of nanoparticle technology and liposomes with biodegradable poly(DL-lactic-co-glycolic acid) (PLGA), cationic surfactant dimethyldioctadecylammonium (DDA) bromide, and the immunopotentiator TDB which promotes Th1 and Th17 CD4^+^ T cell responses and enhanced specific serum antibodies [112]. Moreover, the mycobacterial cell-wall lipid monomycoloyl glycerol analog has been used in combination with DDA. This combination resulted in a promising vaccine delivery system that induced strong antigen-specific Th1 and Th17 responses [113].

Protection against most infections requires both antibodies and adequate T cell immunity. However, for most infectious diseases we have given strongest attention to antibodies as correlates of protection. However, this scenario may change when we will identify more and more T cell mediated parameters that correlate with protection. Recently, it was identified that in influenza the best correlate of protection in a study with healthy human volunteers was the preexisting influenza-specific CD4+T cells [114]. It has been found that high density antigen coating onto liposomes often stimulates better antibody responses than encapsulated antigens, but a combination, such as the DOTAP-PEG-mannose liposomes (LP-Man), will enhance not only antibody responses but also APC antigen uptake and strong memory CD4^+^ T cell development [115–118]. This and other progress in the field have to be considered when developing programs for the evaluation of vaccine efficacy. For example, with liposome vaccines as novel universal, broadly protective, influenza vaccines against also heterosubtypic virus strains, it will be important to also assess and evaluate CD4^+^ T cell immunity as a correlate of protection. Therefore, we need to better define how specific CD4^+^ T cells, and, for that matter CD8^+^ T cells, correlate to impaired viral replication or bacterial growth and reduced transmission of infection.

## 5. Concluding Remarks and Future Perspectives

Here, we have described and discussed recent progress in nanoparticle formulations using liposomes for mucosal vaccine delivery against infectious diseases. We have underscored the complexity of the liposome formulation and pointed to many combinations and plasticity of the liposome nanoparticle as a carrier of vaccine antigens and adjuvants. Needless to say, when considering liposome-based vaccines, it is important to consider all the properties of the formulation, the route of administration, and the biological response. Thus, liposome size, lamellarity, and surface charge as well as lipid composition and fluidity of the membrane can all influence the immune response following vaccination. Importantly, the choice of antigen, with its own inherent physicochemical properties, and the position of the antigen and the adjuvant in the liposome critically affect the function of the liposome. The antigen/lipid ratio and properties of the added adjuvant are also important parameters that change the immunogenicity and stability of the liposome.

Most studies using liposome-based vaccines have focused on the end result of immunization, namely, the magnitude and the quality of the immune response. Few studies have attempted to systematically identify mechanism of action at the different stages of an immune response. For mucosal immunizations liposome vaccines have been thoroughly investigated for their stability in intestinal fluids, their mucoadhesive properties, or the efficiency in targeting and uptake by DCs and other APCs [46, 49, 58, 79, 119, 120]. The site of liposomal penetration of the mucosal barrier is important to determine. If occurring at the duodenal site, the outcome may be significantly different from penetration that takes place in the ileum or colon of the gut. Similarly, antigen uptake in the NALT may be more effective than if the liposome reaches deeper into the respiratory tract. This is not just because of the stability of the liposomal antigen at a distinct location, but also the DC subsets that are exposed to the antigen may differ dramatically and, hence, the outcome of the vaccination may vary. At the cellular level, we largely lack studies that investigate how liposome delivered antigens are processed and presented by DCs, the kinetics of these processes, and whether the formulation will affect the migration and function of the DC in the draining lymph node. More work is needed to generalize the principles for an optimal design of the liposome in this regard. For example, it still remains unclear whether liposomes that rapidly penetrate the mucosal barrier are also good inducers of a mucosal immune response or, alternatively, should mucoadhesive liposomes be used to provide a depot of antigen for an extended loading of DCs with antigen. Simple screening systems should be developed to address these questions. To assess whether a liposome efficiently delivers peptide for CD8+ or CD4+ T cell priming, one could focus on surface expression on the DC of complexes with MHC class I and II molecules plus peptide using specific antibodies that detect these complexes and flow cytometry analysis [121]. In this way, screening of liposome-antigen formulations could be evaluated on the basis of effective expression of such complexes on the surface of a target DC population in vitro and in vivo. The density of such complexes most likely will relate to the ability of the DC to effectively prime the T cells in the lymph node as the T cell receptor does not recognize the peptide, but the complex.

Identifying and standardizing liposome-stimulated immune responses would greatly aid in the prospects of comparing different liposome vaccine formulations for their efficacy. This would also contribute to a more rational design of effective liposome-based mucosal vaccines. Currently, there is no agreed protocol or procedure on how to evaluate and characterize the immune response to liposomes. Hence, most studies are performed without relevant comparisons and the evaluation of whether the liposome formulation is more or less effective compared to other types of formulations is impossible to assess. Therefore, it would be an advantage if investigators could agree on using some standardization protocol, perhaps using cholera toxin or some other strong soluble adjuvant, to admix with antigen and compare the immune response to those of the liposomes. Thus, it is fair to say that we still lack comprehensive comparisons to other modes of formulating antigens for mucosal vaccination.

The liposome technology applied to the development of the next generation of mucosal vaccines holds much promise. It is conceivable that better targeted liposomes with added adjuvant capacity will prove to be effective in stimulating mucosal immune responses that are protective against multiple infectious diseases. It is likely that these liposomes have both surface anchored antigen and encapsulated antigen to optimize B cell as well as T cell priming. The lipid compositions that yield higher gel-liquid crystal transition temperatures will be preferred as they have been found to stimulate stronger immune responses. Cationic rather than neutral liposomes, being more proinflammatory, will be selected for mucosal immunization. It will be important in the future to apply the better understanding of the liposome manufacturing technology and the principles for induction of mucosal immune responses to the design and development of the next generation of mucosal vaccines.

## Figures and Tables

**Table 1:** Examples of liposome adjuvant vaccines against infectious diseases tested in clinical trials. This compilation of completed or ongoing studies involving liposomes for vaccination of humans was generated with data from ClinicalTrials.gov. Here we have indicated the target disease, the vaccine composition, the route of administration, the clinical testing stage, and the reference number.

Name	Disease	Description	Route of administration	Sponsor	Status	ClinicalTrials.gov Identifier
FMP012 with AS01B	Malaria	Falciparum malaria protein (FMP012) in a formulation based on liposomes mixed with the immunostimulants monophosphoryl lipid (MPL) and *Quillaja saponaria* Molina, fraction 21	Intramuscular injection	US Army Medical Research and GlaxoSmithKline	Phase 1	NCT02174978

TVDV with Vaxfectin [21]	Dengue fever	Tetravalent dengue vaccine (TVDV) with Vaxfectin® cationic lipid-based adjuvant	Intramuscular injection	US Army Medical Research and Materiel Command	Phase 1	NCT01502358

Ag85B-ESAT-6 with CAF01 [22]	Tuberculosis	Subunit protein antigen Ag85B-ESAT-6 with two-component liposomal adjuvant system composed of a cationic liposome vehicle (dimethyldioctadecylammonium (DDA) stabilized with a glycolipid immunomodulator (trehalose 6,6-dibehenate (TDB))	Intramuscular injection	Statens Serum Institut	Phase 1	NCT00922363

Biocine with lipid A [23]	HIV	Recombinant envelope protein rgp120/HIV-1SF2 combined with lipid A	Intradermal	National Institute of Allergy and Infectious Diseases	Phase 1	NCT00001042

PAMVAC with GLA-SE or GLA-LSQ	Malaria	Placental malaria vaccine candidate adjuvant with alhydrogel, glucopyranosyl lipid adjuvant-stable emulsion (GLA-SE), or glucopyranosyl lipid adjuvant-liposome-QS-21 formulation (GLA-LSQ)	Intramuscular injection	Tuebingen University Hospital	Phase 1	NCT02647489

ID93 with GLA-SE [24]	Tuberculosis	Recombinant fusion protein incorporating four *M. tuberculosis* antigens (ID93) formulated with glucopyranosyl lipid adjuvant- (GLA-) stable emulsion (SE)	Intramuscular injection	National Institute of Allergy and Infectious Diseases	Phase 1	NCT02508376

Novel liposomal based intranasal influenza vaccine	Influenza	Liposomal-based influenza vaccine	Intranasal	Hadassah Medical Organization	Phase 2	NCT00197301

VaxiSome with CCS/C [25]	Influenza	Commercial split influenza virus and polycationic liposome as adjuvant (CCS/C)	Intramuscular injection	NasVax Ltd.	Phase 2	NCT00915187

Fluzone with JVRS-100 [26]	Influenza	Inactivated trivalent influenza virus vaccine administered with cationic lipid-DNA complex adjuvant JVRS-100	Intradermal	Colby Pharmaceutical Company and Juvaris BioTherapeutics	Phase 2	NCT00936468

RTS/S with AS01 [27]	Malaria	Repeat sequences of the *Plasmodium falciparum* circumsporozoite protein (RTS/S) fused to the hepatitis B surface antigen with a liposome-based adjuvant system that also contains monophosphoryl lipid A (MPL) and *Quillaja saponaria* Molina, fraction 21	Intramuscular injection	KEMRI-Wellcome Trust Collaborative Research Program and GlaxoSmithKline	Phase 3	NCT00872963

Amphomul [28]	Visceral leishmaniasis	Amphotericin B lipid emulsion	Intramuscular injection	Bharat Serums and Vaccines Limited	Phase 3	NCT00876824
